# The association between weight-adjusted-waist index and cognitive function: the cross-sectional and longitudinal evidence from CHARLS

**DOI:** 10.3389/fnut.2025.1601541

**Published:** 2025-10-30

**Authors:** Sifan Qian, Qiuqing Wen, Di Jiang, Shiliang Wang, Xuqiang Hu

**Affiliations:** ^1^Department of Mental Health Prevention and Control, Huzhou Third Municipal Hospital, The Affiliated Hospital of Huzhou University, Huzhou, Zhejiang, China; ^2^Department of Medicine, Huzhou Third Municipal Hospital, The Affiliated Hospital of Huzhou University, Huzhou, Zhejiang, China; ^3^Department of Psychiatric, Huzhou Third Municipal Hospital, The Affiliated Hospital of Huzhou University, Huzhou, Zhejiang, China

**Keywords:** weight-adjusted waist index, obesity, cognitive decline, risk factor, middle-aged and older adults

## Abstract

**Objective:**

Weight-adjusted waist index (WWI), a novel indicator of abdominal obesity that reflects body compositional changes due to aging, shows superiority in predicting obesity-related health risks. The present study aimed to investigate the cross-sectional and longitudinal association between WWI and cognitive function in the Chinese middle-aged and older population.

**Methods:**

A total of 8,822 individuals were included in cross-sectional analyses, of whom 7,697 had longitudinal data. The baseline WWI was calculated as waist circumference divided by the square root of body weight. Cognitive scores were assessed at baseline and every 2–3 years during follow-up. The primary outcome was cognitive z-scores. Linear regression and generalized estimating equation models were used to assess the cross-sectional association of WWI with cognitive function and its longitudinal association with cognitive decline, respectively.

**Results:**

Higher WWI was associated with lower baseline global cognitive score (β = −0.218 per unit increase; 95% CI: −0.333 to −0.103). Longitudinally, there was a significant association between WWI and accelerated global cognitive decline (β = −0.008 per unit increase; 95% CI: −0.013 to −0.003). However, higher body mass index was associated with slower cognitive decline, whereas waist circumference showed no significant association with cognitive decline.

**Conclusion:**

Higher baseline WWI levels were independently associated with an increased risk of subsequent cognitive decline, indicating WWI may be a potential obesity indicator for identifying poor cognitive performance among middle-aged and older adults.

## 1 Introduction

Cognitive decline is a major manifestation of aging that has been regarded as a precursor of mild cognitive impairment (MCI) or dementia, posing huge burden of health and economic on families and society (1, 2). In China, the population has aged rapidly, dementia and MCI are highly prevalent in older adults with the prevalence of 6.0% (15 million) and 15.5% (38.8 million), respectively (3). Since there are no effective treatments available for dementia, practical indicators are warranted for early identification of cognitive decline population and implementing targeted measures to prevent its progression to MCI or dementia.

Obesity, with the increasing prevalence worldwide, has been established as a risk factor for a variety of chronic medical conditions (such as diabetes and cardiovascular diseases) that are associated with poor cognitive performance and dementia (4–6). However, current evidence on the associations between traditional adiposity indices and cognitive function remains inconsistent (7–12). For example, Zeng et al. found that raised body mass index (BMI) and waist circumference (WC) at midlife significantly increased the subsequent risk of dementia among Chinese adults (9). While a large cohort study of Chinese hypertensive adults reported that BMI-based general adiposity and WC-based abdominal adiposity were both correlated with slower cognitive decline, a counterintuitive finding that has been termed the “obesity paradox” in cognitive aging (11). This paradox could be partly attributed to inherent limitations of BMI and WC, which fail to differentiate between lean and fat mass as well as between subcutaneous adipose tissue and visceral adipose tissue (13). Therefore, other effective obesity indices that provide accurate estimation of the distribution of fat accumulation and free from obesity paradox are clearly needed.

Weight-adjusted waist index (WWI), a novel central obesity metric that combines the strengths of waist circumference while attenuating its correlation with BMI, has been proposed to alleviate the obesity paradox (14). In addition, several imaging studies have shown that WWI effectively captures aging-related body-compositional changes in middle-aged and older adults and outperforms traditional measures like BMI and WC (15–17). Emerging evidence have shown significant relationships of WWI and cardiometabolic risk factors and diseases, all of which are implicated in poor cognitive performance (18–20). Recently, several cross-sectional studies conducted in American older adults suggested a positive association of WWI and low cognitive score, which was stronger than the association between BMI and WC (21–23). Similar results were found in rural Chinese older adults (24). However, these studies were all cross-sectional, providing limited information on temporality, and their participants were mainly American or Chinese older adults, limiting the generalizability of the findings.

Therefore, we investigated the cross-sectional and longitudinal associations of WWI with cognitive function using the data from the China Health and Retirement Longitudinal Study (CHARLS).

## 2 Materials and methods

### 2.1 Study participants

The study participants were recruited from the CHARLS, the design details have been described previously (25). In brief, the CHARLS is a nationally representative ongoing population-based prospective study of community-dwellers aged ≥45 years from 450 villages within 28 provinces in China, which was designed to comprehensively understand the socioeconomic determinants and consequences of aging. At wave 1 (baseline survey), 17,708 respondents were recruited and they are followed every two or three years.

**Figure 1:**
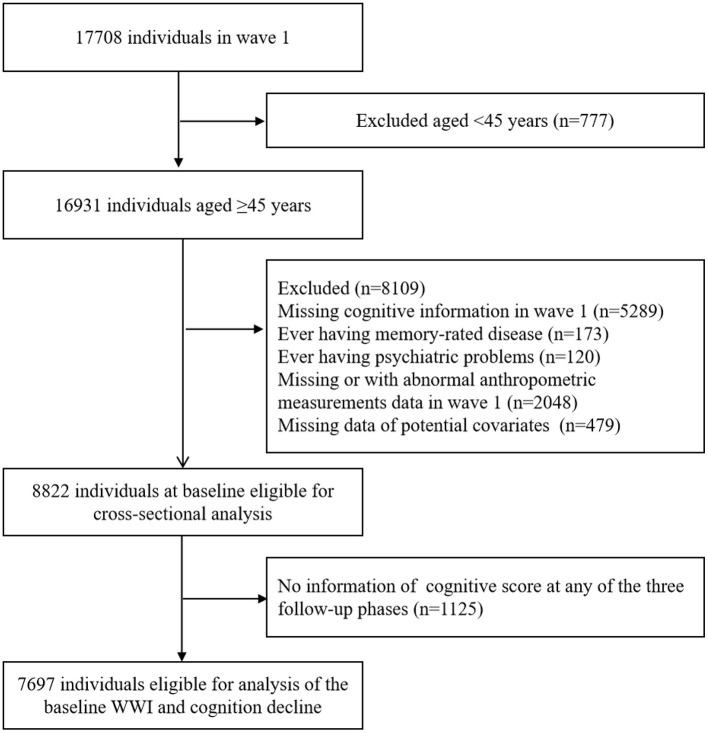
Flowchart of the study. WWI indicates weight-adjusted waist index.

In the present study, we used data from four waves collected in 2011 (wave 1), 2013 (wave 2), 2015 (wave 3) and 2018 (wave 4). The sample size in wave 1 was 17,708. The exclusion criteria were as follows: (1) age less than 45 years; (2) without cognitive information in wave 1; (3) ever having memory-rated disease; (4) ever having psychiatric problems; (5) without or with abnormal value of anthropometric measurements data in wave 1; (6) missing data of potential covariates. The cross-sectional analysis included 8,822 participants. In the longitudinal analysis, we further excluded the participants with no information on cognitive function at any of the three follow-up phases. Finally, a total of 7,697 individuals were eligible for the analysis of baseline WWI and cognitive decline (Figure 1).

The CHARLS protocol was approved by the Ethical Review Committee of Peking University (IRB00001052-11015). All study participants provided written informed consent.

### 2.2 Anthropometric measurements

Anthropometric measurements, including height, weight and WC, were assessed by trained staff in accordance with the standard procedures. The stadiometer and a weighing scale were used to measure height (unit: cm) and body weight (unit: kg) with light clothes and no shoes. WC (unit: cm) was assessed in the standing position by placing the soft measuring tape over the clothing around the waist at the level of the navel. The definition of abdominal obesity was based on a value of WC ≥90 cm for male and ≥85 cm for female (26). WWI was calculated as WC divided by the square root of weight (cm/√kg) (14). BMI was calculated as weight divided by height squared (kg/m^2^), and was categorized into four groups: underweight (< 18.5 kg/m^2^), normal weight (18.5–23.9 kg/m^2^), overweight (24–27.9 kg/m^2^) and obesity (≥28 kg/m^2^) (27).

### 2.3 Cognitive function assessment

Cognitive function was measured from two domains (episodic memory and executive function). The first domain was episodic memory, which included immediate word recall and delayed word recall, with scores ranging from 0 to 20 points. The content and administration of word recall were consistent in the first three waves. In wave 4, CHARLS introduced the Harmonized Cognitive Assessment Protocol to align its cognitive measures with other aging surveys, updating the word list and the number of immediate- and delayed-recall trials (28). To address these changes, a weighted equipercentile equating method was used to adjust the word recall scores in wave 4 (29). The second domain was executive function, which comprised of three measurements: orientation (score range, 0–5 points), computation (score range, 0–5 points), and pentagon drawing test (score range, 0–1 points). After summing the scores of these three measurements, the total score of executive function ranged from 0 to 11 points. Baseline global cognitive function scores were defined by the total of these two domains, with a scale varying from 0 to 31 points, and higher scores indicated better cognitive performance.

To enable longitudinal comparability across cognitive tests, a standardized z-score for each of the two cognitive domains (episodic memory and executive function) was calculated by subtracting the baseline mean and dividing by the baseline SD. The global cognitive z-score was derived by averaging the two domain-specific z-scores and then re-standardizing this using baseline values of the global cognitive *z*-scores (30, 31).

### 2.4 Covariates

The covariates in our study included demographic characteristics (age, sex, residence, marital status, education level and working status), lifestyle factors (smoking and drinking status, social participation, and sleep duration) and health conditions (hearing status, viewing problems, medical history [hypertension, diabetes, stroke, cancer], medication use history, Center for Epidemiologic Studies Depression [CES-D] Scale score, restriction on basic activities of daily living [BADL]) and handgrip strength. Details of covariates definition can be found in eMethods in the Supplementary material.

### 2.5 Statistical analysis

All study participants were categorized according to sex specific quartiles of baseline WWI. Continuous variables were described by median with interquartile range (IQR) due to the non-normal distribution. And the Mann–Whitney U-tests was conducted to test for trend across the quartiles of WWI. Categorical variables were presented as frequency with percentile, and the chi-squared test was conducted to assess for trend across the quartiles.

We explored the cross-sectional association between WWI and cognitive performance, as well as the longitudinal relationship between WWI and subsequent cognitive decline. First, linear regression models were performed to test the association between WWI and cognitive score at baseline, with calculating regression coefficients (β) and corresponding 95% confidence intervals (CIs). Second, accounting for repeated cognitive assessments, we applied generalized estimating equations with an exchangeable working correlation structure to assess the association between baseline WWI and cognitive decline. An interaction term of WWI × follow-up time (year) was added in the models, its β coefficients indicated the mean differences in annual rates of cognitive decline per unit increase in WWI or across WWI categories, with positive values indicating decelerated decline and negative values indicating accelerated decline. The multivariable-adjusted models were further controlled for age, sex, marital status, residence, educational level, working status, smoking status, drinking status, sleep hours, social participation, medical history (hypertension, diabetes, stroke and cancer), medication history (antihypertensive medications and glucose-lowering medications), hearing status, viewing problems, CESD-score, restriction on BADL and handgrip strength. Similar analyses were repeated when examined the association between BMI and WC in relation to baseline cognitive score and cognitive decline.

Given the influence of age, sex and educational level on cognitive function (31, 32), the interaction between WWI and age, sex, as well as educational level, on cognitive decline was examined separately. Stratified analyses were carried out to examine whether the associations of baseline WWI with cognitive decline differed according to age (< 60 vs. ≥60 years), sex (male vs. female) and education level (primary school or below vs. junior high school or above).

Furthermore, sensitivity analyses were conducted to validate the robustness of results: (1) excluding those with stroke at baseline; (2) using unstandardized cognitive function scores as outcomes. Statistical analysis was conducted using R software (version 4.3.0). All *P*-values were 2-tailed, and *P*-values < 0.05 was considered to be statistically significant.

## 3 Results

### 3.1 Baseline characteristics

There were 8,822 participants (4,623 male and 4,199 female) with a median age (IQR) of 57.0 (51.0–64.0) years included in the cross-sectional analysis. Compared to participants in the lowest quartile of baseline WWI, those in higher WWI levels were more likely to be older, unmarried, live in the urban, be illiteracy, sleep less; to have higher CESD score, BMI and WC; to have a lower proportion of working; to have higher prevalence of restriction on BADL, poorer hearing and viewing, hypertension, diabetes, stroke, antihypertensive and glucose-lowering medications use; to have lower levels of handgrip strength; while were less likely to be current cigarette smokers (Table 1).

**Table 1:** Baseline characteristics according to sex-specific quartiles of baseline weight-adjusted-waist index (*N* = 8,822).

**Characteristics^a^**	**Baseline weight-adjusted-waist index, cm/**√**kg**				***P*-value**
	**Q1**	**Q2**	**Q3**	**Q4**	
Number of participants	2,206 (25.01)	2,208 (25.03)	2,205 (24.99)	2,203 (24.97)	
**Demographic**					
Age, *y*	54.0 (48.0–60.0)	56.0 (50.0–62.0)	58.0 (52.0–64.0)	62.0 (56.0–69.0)	<0.001
Male, *n* (%)	1,156 (52.4)	1,156 (52.4)	1,157 (52.5)	1,154 (52.4)	1
Married (vs others), *n* (%)	2,015 (91.3)	2,002 (90.7)	1,965 (89.1)	1,879 (85.3)	<0.001
Rural residence (vs urban), *n* (%)	1,446 (65.5)	1,360 (61.6)	1,282 (58.1)	1,236 (56.1)	<0.001
Educational level, *n* (%)					<0.001
Illiterate	348 (15.8)	391 (17.7)	444 (20.1)	564 (25.6)	
≤Primary school	897 (40.7)	927 (42.0)	931 (42.2)	1,027 (46.6)	
Junior high school	576 (26.1)	576 (26.1)	556 (25.2)	410 (18.6)	
High school and above	385 (17.4)	314 (14.2)	274 (12.4)	202 (9.2)	
Working, *n* (%)	1,663 (75.4)	1,548 (70.1)	1,471 (66.7)	1,227 (55.7)	<0.001
**Lifestyle**, ***n*** **(%)**					
Smoking status					<0.001
Never	1,239 (56.1)	1,261 (57.1)	1,268 (57.5)	1,242 (56.4)	
Former	167 (7.6)	200 (9.1)	227 (10.3)	266 (12.1)	
Current	800 (36.3)	747 (33.8)	710 (32.2)	695 (31.5)	
Drinking status					0.12
Never	1,341 (60.8)	1,253 (56.8)	1,270 (57.6)	1,291 (58.6)	
<1 time/month	177 (8.0)	199 (9.0)	186 (8.4)	168 (7.6)	
≥1 time/month	688 (31.2)	756 (34.2)	749 (34.0)	744 (33.8)	
Social participation, *n* (%)	1,072 (48.6%)	1,094 (49.5%)	1,088 (49.3%)	1,083 (49.2)	0.93
Sleep duration (hours)					0.001
≤6	1,035 (46.9)	1,086 (49.2)	1,083 (49.1)	1,163 (52.8)	
6–8	1,016 (46.1)	949 (43.0)	951 (43.1)	859 (39.0)	
>8	155 (7.0)	173 (7.8)	171 (7.8)	181 (8.2)	
**Health conditions**, ***n*** **(%)**					
Hearing status					<0.001
Very good	424 (19.2)	326 (14.8)	354 (16.0)	323 (14.6)	
Good	696 (31.6)	689 (31.2)	643 (29.2)	637 (28.9)	
Fair	867 (39.3)	957 (43.3)	961 (43.6)	964 (43.8)	
Poor	219 (9.9)	236 (10.7)	247 (11.2)	279 (12.7)	
Viewing problem	1,642 (74.4)	1,708 (77.4)	1,689 (76.6)	1,754 (79.6)	0.001
**Medical history**					
Hypertension	612 (27.7)	785 (35.6)	961 (43.6)	1,210 (54.9)	<0.001
Diabetes	162 (7.3)	247 (11.2)	327 (14.8)	405 (18.4)	<0.001
Stroke	39 (1.8)	40 (1.81)	47 (2.1)	63 (2.9)	0.045
Cancer	14 (0.6)	25 (1.1)	17 (0.8)	22 (1.0)	0.29
**Medication history**					
Use of antihypertensive medications	274 (12.4)	344 (15.6)	414 (18.8)	611 (27.7)	<0.001
Use of glucose-lowering medications	38 (1.7)	76 (3.44)	95 (4.3)	159 (7.2)	<0.001
CESD score	6.0 (3.0–11.0)	6.0 (3.00–11.0)	6.0 (3.0–11.0)	7.0 (3.0–12.0)	0.006
Restriction on BADL	197 (8.9)	223 (10.1)	327 (14.8)	390 (17.7)	<0.001
Handgrip strength (kg)	34.0 (27.9–41.5)	33.0 (27.0–41.0)	32.0 (26.0–40.0)	30.0 (23.5–38.0)	<0.001
Body mass index (kg/m^2^)	21.7 (20.0–23.7)	22.9 (20.8–25.0)	24.0 (21.7–26.5)	24.4 (21.9–26.9)	<0.001
Waist circumstance (cm)	76.2 (72.0–81.0)	83.0 (78.2–88.0)	88.2 (83.0–93.2)	93.0 (87.0–99.2)	<0.001

BADL, basic activities of daily living; CESD, Center for Epidemiological Studies Depression Scale;

^a^Continuous variables are expressed as median (interquartile range). Categorical variables are expressed as frequency (percent).

The three adiposity indices were significantly correlated with each other (*P* < 0.001) (Supplementary files, Supplementary Table 1). WC was highly correlated with BMI (correlation coefficients: 0.810, *P* < 0.001), whereas WWI was less strongly correlated with BMI (correlation coefficients: 0.260, *P* < 0.001).

### 3.2 Cross-sectional association between adiposity indices and cognitive function

Table 2 presented the cross-sectional association between adiposity indices and cognitive score at baseline. In the multivariable linear regression model, higher WWI levels were significantly associated with lower scores in cognitive function (global cognitive function, β = −0.218 per 1 unit increase in WWI, *P* < 0.001; episodic memory, β = −0.153 per 1 unit increase in WWI, *P* < 0.001; executive function, β = −0.065 per 1 unit increase in WWI, *P*=0.029). However, elevated levels of BMI and WC were correlated with higher global cognitive scores (both *P* < 0.05).

**Table 2:** Associations of adiposity indices with baseline cognitive function (*N* = 8,822).

**Adiposity Indices**	**Global cognition**		**Episodic memory**		**Executive function**	
	***β*** **(95%CI)**	***P*** **-value**	***β*** **(95%CI)**	***P*** **-value**	***β*** **(95%CI)**	***P-*** **value**
**WWI**			Reference			
Continues	−0.218 (−0.333 to −0.103)	<0.001	−0.153 (−0.240 to −0.066)	<0.001	−0.065 (−0.123 to −0.007)	0.029
Q1	Reference		Reference		Reference	
Q2	−0.071 (−0.305 to 0.163)	0.554	−0.089 (−0.266 to 0.089)	0.328	0.018 (−0.100 to 0.136)	0.765
Q3	−0.209 (−0.446 to 0.029)	0.085	−0.219 (−0.399 to −0.038)	0.017	0.010 (−0.109 to 0.130)	0.868
Q4	−0.306 (−0.555 to −0.057)	<0.016	−0.224 (−0.413 to −0.035)	0.020	−0.083 (−0.208 to 0.043)	0.196
**BMI**						
Continues	0.053 (0.026 to 0.080)	<0.001	0.021 (0.0004 to 0.042)	0.046	0.032 (0.018 to 0.046)	<0.001
Normal weight	Reference		Reference		Reference	
Underweight	−0.291 (−0.661 to 0.079)	0.123	−0.214 (−0.495 to 0.067)	0.136	−0.077 (−0.263 to 0.109)	0.417
Overweight	0.280 (0.084 to 0.475)	0.005	0.083 (−0.066 to 0.231)	0.275	0.197 (0.099 to 0.295)	<0.001
Obesity	0.366 (0.071 to 0.661)	0.015	0.147 (−0.077 to 0.371)	0.199	0.219 (0.070 to 0.367)	0.004
**WC**						
Continues	0.016 (0.007 to 0.025)	<0.001	0.003 (−0.004 to 0.010)	0.370	0.013 (0.008 to 0.017)	<0.001
Normal	Reference		Reference		Reference	
Abdominal obesity	0.292 (0.111 to 0.473)	0.002	0.074 (−0.064 to 0.211)	0.296	0.219 (0.127 to 0.310)	<0.001

The β and P-values are adjusted for age, sex, marital status, residence, educational level, working status, smoking status, drinking status, sleep hours, social participation, medical history (hypertension, diabetes, stroke and cancer), medication history (antihypertensive medications and glucose-lowering medications), hearing status, viewing problems, CESD-score, restriction on BADL and handgrip strength.

### 3.3 Longitudinal association between adiposity indices and cognitive decline

In the longitudinal analyses, higher WWI was significantly associated with accelerated cognitive decline (Table 3). Compared to participants in the lowest quartile of WWI, those in the highest quartile group exhibited accelerated decline in global cognition (β = −0.026, 95% CI: −0.036 to −0.016), episodic memory (β = −0.029, 95% CI: −0.040 to −0.018) and executive function (β = −0.014, 95% CI: −0.024 to −0.004).

**Table 3:** Associations of adiposity indices with cognitive decline (*N* = 7,697).

**Adiposity indices**	**Global cognition**		**Episodic memory**		**Executive function**	
	***β*** **(95%CI)**	***P*** **-value**	***β*** **(95%CI)**	***P*** **-value**	***β*** **(95%CI)**	***P-*** **value**
**WWI**						
Continues WWI × time	−0.008 (−0.013 to −0.003)	0.001	−0.011 (−0.016 to −0.006)	<0.001	−0.004 (−0.008 to 0.001)	0.109
**WWI quartiles** **×time**						
Q1	Reference		Reference		Reference	
Q2	−0.009 (−0.019 to 0.001)	0.065	−0.008 (−0.019 to 0.003)	0.145	−0.004 (−0.013 to 0.006)	0.450
Q3	−0.010 (−0.020 to 0.0004)	0.059	−0.008 (−0.019 to 0.003)	0.163	−0.006 (−0.016 to 0.004)	0.229
Q4	−0.026 (−0.036 to −0.016)	<0.001	−0.029 (−0.040 to −0.018)	<0.001	−0.014 (−0.024 to −0.004)	0.006
**BMI**						
Continues BMI × time	0.002 (0.001 to 0.003)	0.003	0.002 (0.001 to 0.004)	<0.001	0.001 (−0.0001 to 0.002)	0.080
**BMI categories** **×time**						
Normal weight	Reference		Reference		Reference	
Underweight	−0.010 (−0.029 to 0.008)	0.272	−0.011 (−0.029 to 0.008)	0.255	−0.008 (−0.027 to 0.012)	0.446
Overweight	0.004 (−0.004 to 0.012)	0.339	0.010 (0.001 to 0.019)	0.028	0.002 (−0.006 to 0.010)	0.566
obesity	0.008 (−0.004 to 0.020)	0.186	0.014 (0.001 to 0.027)	0.042	0.005 (−0.007 to 0.017)	0.413
**WC**						
Continues WC × time	−0.0001 (−0.0005 to 0.0002)	0.509	0.0003 (−0.0001 to 0.0007)	0.192	−0.0002 (−0.0006 to 0.001)	0.199
**WC categories** **×time**						
Normal	Reference		Reference		Reference	
Abdominal obesity	−0.002 (−0.009 to 0.005)	0.509	0.002 (−0.005 to 0.010)	0545	−0.003 (−0.011 to 0.004)	0.367

The β and P-values are adjusted for age, sex, marital status, residence, educational level, working status, smoking status, drinking status, sleep hours, social participation, medical history (hypertension, diabetes, stroke and cancer), medication history (antihypertensive medications and glucose-lowering medications), hearing status, viewing problems, CESD-score, restriction on BADL and handgrip strength.

In contrast, a higher BMI was associated with decelerated cognitive decline. Compared to normal weight, the overweight showed a slower decline in episodic memory (β = 0.010, 95% CI: 0.001 to 0.019), but no significant differences in the rate of change for global cognitive function (β = 0.004, 95% CI: −0.004 to 0.012) or executive function (β = 0.002, 95% CI: −0.006 to 0.010). Similar results were also found in the obesity group. In addition, WC was not significantly associated with cognitive decline in either the continues or the category.

### 3.3 Subgroup and sensitivity analyses

No significant effect modification by age, sex, or educational level on the association between baseline WWI and cognitive decline (*P*-interaction > 0.05 for all) was observed. In most strata, higher baseline WWI levels were significantly associated with cognitive decline (Table 4).

**Table 4:** Subgroup analyses of the association between WWI with the risk of global cognition decline.

**Characteristics**	**Continues WWI × time**	**WWI quartiles** × **time**				***P*-value for interaction**
		**Q1**	**Q2**	**Q3**	**Q4**	
**Age**						0.505
<60	−0.003 (−0.009 to 0.003)	Reference	−0.009 (−0.020 to 0.003)	−0.009 (−0.021 to 0.003)	−0.022 (−0.036 to −0.009)	
≥60	−0.007 (−0.014 to −0.0002)	Reference	−0.004 (−0.023 to 0.014)	0.001 (−0.017 to 0.019)	−0.012 (−0.029 to 0.005)	
**Sex**						0.584
Male	−0.011 (−0.018 to −0.004)	Reference	−0.009 (−0.022 to 0.004)	−0.006 (−0.019 to 0.008)	−0.020 (−0.033 to −0.006)	
Female	−0.014 (−0.020 to −0.007)	Reference	−0.009 (−0.024 to 0.005)	−0.014 (−0.029 to 0.001)	−0.034 (−0.050 to −0.018)	
**Educational level**						0.705
≤Primary school	−0.005 (−0.011 to 0.001)	Reference	−0.004 (−0.019 to 0.011)	−0.007 (−0.021 to 0.008)	−0.019 (−0.033 to −0.005)	
≥Junior high school	−0.006 (−0.013 to 0.001)	Reference	−0.012 (−0.025 to 0.001)	−0.010 (−0.024 to 0.003)	−0.027 (−0.042 to −0.012)	

The P-value of the interaction term between WWI, time with age or sex or educational level, respectively. Analyses were also stratified by age, sex and educational level. The β is adjusted for age, sex, marital status, residence, educational level, working status, smoking status, drinking status, sleep hours, social participation, medical history (hypertension, diabetes, stroke and cancer), medication history (antihypertensive medications and glucose-lowering medications), hearing status, viewing problems, CESD-score, restriction on BADL and handgrip strength, except for the stratified variable.

The association between baseline WWI and cognitive decline was not substantial changed after excluding participants with stroke at baseline. Furthermore, findings were consistent with the main analyses when using the unstandardized score analyses for cognitive function (Table 5).

**Table 5:** Associations of WWI with cognitive decline-sensitivity analysis.

**Adiposity Indices**	**Global cognition**		**Episodic memory**		**Executive function**	
	***β*** **(95%CI)**	***P*** **-value**	***β*** **(95%CI)**	***P*** **-value**	***β*** **(95%CI)**	***P-*** **value**
**Excluding individuals with a history of stroke (*****N** **=*** **7,543)**						
Continues WWI × time	−0.008 (−0.013 to −0.003)	0.002	−0.011 (−0.016 to −0.006)	<0.001	−0.004 (−0.008 to 0.001)	0.116
**WWI quartiles** **×time**						
Q1	Reference		Reference		Reference	
Q2	−0.009 (−0.019 to 0.001)	0.079	−0.008 (−0.019 to 0.003)	0.179	−0.003 (−0.013 to 0.006)	0.504
Q3	−0.010 (−0.020 to 0.0004)	0.060	−0.008 (−0.020 to 0.003)	0.144	−0.006 (−0.016 to 0.004)	0.277
Q4	−0.026 (−0.036 to −0.015)	<0.001	−0.028 (−0.040 to −0.017)	<0.001	−0.014 (−0.024 to −0.004)	0.007
**Using unstandardized scores**						
Continues WWI × time	−0.037 (−0.059 to −0.016)	<0.001	−0.035 (−0.051 to −0.019)	<0.001	−0.008 (−0.019 to 0.002)	0.109
**WWI quartiles** **×time**						
Q1	Reference		Reference		Reference	
Q2	−0.046 (−0.092 to −0.0004)	0.048	−0.026 (−0.062 to 0.009)	0.145	−0.008 (−0.030 to 0.013)	0.450
Q3	−0.045 (−0.091 to 0.002)	0.059	−0.026 (−0.061 to 0.010)	0.163	−0.014 (−0.036 to 0.009)	0.229
Q4	−0.123 (−0.170 to −0.075)	<0.001	−0.094 (−0.130 to −0.058)	<0.001	−0.032 (−0.055 to −0.009)	0.006

The β and P-values are adjusted for age, sex, marital status, residence, educational level, working status, smoking status, drinking status, sleep hours, social participation, medical history (hypertension, diabetes, stroke and cancer), medication history (antihypertensive medications and glucose-lowering medications), hearing status, viewing problems, CESD-score, restriction on BADL and handgrip strength.

## 4 Discussion

In this nationwide, prospective cohort of Chinese middle-aged and older adults, we demonstrated a baseline cross-sectional association between WWI and cognitive score. In addition, higher baseline WWI was associated with greater cognitive decline during follow-up, independently of demographic characteristics, lifestyle factors and health conditions. In contrast, higher BMI was correlated with a decelerated cognitive decline, whereas WC showed no significant longitudinal association. These findings indicated that WWI could be a promising obesity predictor for the early identification of poor cognitive performance, and that evaluating WWI may aid in predicting future cognitive decline risk among middle-aged and older adults.

Accumulating studies have investigated the relationship between obesity and cognitive function and yielded inconsistent results (7–12). For instance, Xu et al. found that both BMI-defined overweight and obesity at midlife independently increased the risk of dementia in later life among Swedish twin individuals (7). In contrast, a retrospective cohort study involving nearly 2 million people from the UK reported that being overweight or obesity, measured by BMI, in middle and old age was associated with a decreased odds ratio of dementia, the phenomenon termed as the “obesity paradox” (10). Similarly, we support the inverse relationship between BMI and cognitive decline in Chinese middle-aged and older adults. Although WC more precisely reflects abdominal fat accumulation, the “obesity paradox” persists when adiposity is quantified using WC. Specifically, a prior cohort study revealed that WC was associated with slower cognitive decline in Chinese hypertensive adults (11). In our study, abdominal obesity defined by WC was cross-sectional associated with higher cognitive scores, whereas longitudinal analyses revealed no significant association. The discrepancy of results may partly due to the differences in study populations, cognitive assessments, follow-up duration, and adjustment for potential confounders. Overall, current evidence on the association between traditional adiposity indices and cognitive function is still inconclusive.

The mechanisms underlying the negative associations between BMI and WC in relation to cognitive function remains elusive, and several hypotheses have been proposed. In older adults, high BMI may result from higher muscle mass or accumulation of fat in non-abdominal area (12). Indeed, larger leg lean mass enhances glucose metabolism, thereby preventing glucotoxicity (33). A maintained proportion of gynoid fat could help prevent cognitive decline (34). Another possible explanation is “healthy survivor” effect in the sample, whereby individuals with higher BMI who have reached older ages are healthier, whereas those at greatest risk have already died and are therefore absent from the sample. On the other hand, the “obesity paradox” may not actually exist, possibly due to the fact that BMI fails to differentiate fat mass and muscle mass, leading to the misclassification of obesity status (13). For example, a recent study identified individuals with BMI below 30 kg/m^2^ but markedly elevated WWI, who would traditionally be considered non-obese (35). Moreover, WC is strongly correlated with BMI, and could not differentiate subcutaneous adipose tissue and visceral adipose tissue (13). Further research is needed to elucidate underlying pathophysiological mechanisms.

WWI, a novel abdominal obesity indicator that reflects unfavorable aging-related body compositional changes, has exhibited its superiority in predicting obesity-related health risks (14, 18–20). Currently, some researches have explored the relationship between WWI and the risk of dementia or cognitive impairment. Evidence from the National Health and Nutrition Examination Survey described a cross-sectional association between elevated WWI and lower cognitive score among older U.S. adults (24–26). Similarly, in the MIND-China Study of 5,277 rural older adults, Wang et al. demonstrated a linear association between WWI and both all-cause dementia and Alzheimer's dementia, with WWI outperforming BMI and WC (24). In line with these findings, our cross-sectional analysis supports an inverse association between WWI and cognitive performance. Nevertheless, the existing literature is entirely cross-sectional and limited to older adults, which restricted the generalization of the findings. Our prospective study from the nationally representative CHARLS suggested the longitudinal association between WWI and cognitive decline, extending the current evidence. If our results are further confirmed, WWI might be useful in risk stratification of cognitive decline, and keeping the WWI within an appropriate level might be beneficial for the prevention of cognitive decline.

Several underlying mechanisms could explain the relationship between WWI and cognitive impairment. Firstly, epidemiology studies demonstrated that elevated WWI levels were associated with cardiovascular risk factors and cardiovascular diseases (18–20), which are confirmed to have detrimental effects on cognitive performance (36). Secondly, as WWI is positively associated with fat mass and negatively associated with muscle mass, higher WWI could represent the status of sarcopenic obesity (16). Population-based studies have reported that sarcopenic obesity was associated with worse brain structure, cognitive impairment and dementia (37, 38). And the possible mechanisms were inflammation, insulin resistance, hormone, and leptin (39, 40). Thirdly, WWI was found to be linearly positively correlated with serum neuroflament light protein, which is thought to reflect the neuronal injury and is associated with cognitive decline (41).

Several limitations of the current study warrant consideration. First, the causal association between WWI and cognitive function could not be established due to the inherent limitations of observational study. Second, WWI could be influenced by exercise and nutrition (42). However, nearly 60% of physical activity values were missing, and information on dietary patterns was not available. Therefore, the confounding effect could not be examined in the present study due to missing data. Instead, handgrip strength, a surrogate indicator that concurrently reflects nutritional status and overall physical performance (43), were included to the multivariable models. Future studies that incorporate dietary questionnaires and physical activity assessments will be required to comprehensively clarify the relationship between WWI and cognitive decline. Third, WWI is sensitive to measurement errors in both WC and body weight, which can be amplified when the index is calculated. However, WC and body weight were measured by trained staff according to the standard protocol, such errors could be minimized. Fourth, as a newly proposed obesity metric, there is currently no standardized cut-off points for classifying obesity based on WWI, which limits clinical interpretation and cross-study comparisons. Therefore, further analysis in larger and more representative populations is required.

## 5 Conclusion

In this population-based study of Chinese middle-aged and older adults, higher WWI was cross-sectionally associated with poorer cognitive performance and prospectively linked to accelerated cognitive decline. These findings support that WWI could serve as a potential obesity indicator for evaluating the risk of cognitive decline.

## Data Availability

The raw data supporting the conclusions of this article will be made available by the authors, without undue reservation.
