# Supplementary material for: The association between weight-adjusted-waist index and cognitive function: the cross-sectional and longitudinal evidence from CHARLS
Source: Front Nutr. 2025 Oct 30;12:1601541. doi: 10.3389/fnut.2025.1601541 (PMC12611687; doi:10.3389/fnut.2025.1601541)
Supplement: Supplementary file 1 [file Table_1.docx]

**eMethods.** Covariates

The covariates in our study included demographic characteristics, lifestyle factors and health conditions. Demographic characteristics included age (y), sex (male or female), residence (rural or urban), marital status (married or others), education level (illiterate, ≤primary school, junior high school, or high school and above) and working status (yes or no). Lifestyle factors comprised smoking status (never, former, or current), drinking status (never, <1 time/month, or ≥1 time/month), social participation (yes or no), and sleep duration (≤6.0, 6.0-8.0, or >8.0 h). Health conditions consisted of hearing status (very good, good, fair or poor) and viewing problems (yes or no), medical history (hypertension, diabetes, stroke, cancer), medication use history (antihypertensive medications and glucose-lowering medications), depressive status (assessed by 10-item Center for Epidemiologic Studies Depression Scale [CESD]), restriction on BADL (difficulty in performing one or more activities of basic activities of daily living encompassing eating, dressing, getting in/out of bed, using the toilet, bathing, and controlling urination or defecation, yes or no), and handgrip strength.

Supplementary Table 1 Pearson Correlation Coefficients Between Adiposity Indices and Age (n=8822)

| Adiposity indices | WWI | BMI | WC |
| --- | --- | --- | --- |
| WWI | 1 |  |  |
| BMI | 0.260^*^ | 1 |  |
| WC | 0.606^*^ | 0.810^*^ | 1 |

^*^*P* <0 .001

Abbreviations: WWI, weight-adjusted waist index; BMI, body mass index; WC, waist circumference.
